# Mowing Modulates the Biotic Filter of Expansive Species

**DOI:** 10.1002/ece3.72773

**Published:** 2026-01-05

**Authors:** Alessandro Bricca, Giacomo Cangelmi, Arianna Ferrara

**Affiliations:** ^1^ Faculty of Agricultural, Environmental and Food Sciences Free University of Bozen‐Bolzano Italy; ^2^ Department of Life, Health and Environmental Science University of L'Aquila Italy; ^3^ Department of Biological, Geological and Environmental Sciences Alma Mater Studiorum University of Bologna Italy

**Keywords:** community assembly, competition, expansive species, management types, mowing, phylogenetic diversity, semi‐natural grassland

## Abstract

Disturbance and competition are two strong drivers of plant community formation. Disturbances like mowing in semi‐natural grasslands enhance taxonomic and phylogenetic diversity by preventing the establishment of expansive species, which act as a biotic filter. However, how mowing and expansive species influence alpha and beta diversity, and how mowing may modulate the effects of expansive species on plant communities, remains largely overlooked. We sampled 61 (0.5 × 0.5 m) vegetation plots in abandoned and mown semi‐natural grassland in Central Italy characterised by varying degrees of cover of expansive species. We used Rao's Quadratic Entropy to quantify alpha and beta taxonomic and phylogenetic diversity and we performed multiple regression models to evaluate the effect of management types (mowing and abandonment), expansive species cover, and their interaction. Overall, mown grasslands hosted higher alpha and beta taxonomic and phylogenetic diversity than abandoned grasslands. Alpha taxonomic and phylogenetic diversity decreased with increasing expansive species cover, whereas for beta diversity, only the taxonomic facet is negatively affected. For all diversities except beta‐phylogenetic diversity, we detected a significant interaction between the effect of management types and expansive species cover, with their effects being stronger in mown than abandoned grasslands. Mowing represents a management type capable of counteracting homogenisation due to land abandonment at multiple spatial scales, by allowing a higher number of species with distinct lineages to persist locally. Expansive species acts as a biotic filter in reducing the plant diversity within the community, but its effect on homogenising plant communities was weaker. However, the intensity of this biotic filter was more intense in mown grasslands than in abandoned ones, probably due to higher species richness. Our findings highlight how complex these drivers are and that management can modulate the filterering effect of expansive species.

## Graphical abstract

Management type and expansive species influence plant community diversity in complex ways. Overall increase of expansive species cover acts as biotic filter in reducing the alpha and beta taxonomic and phylogenetical diversity. However, the filtering effect is modulated by management type, being more intense under mown grasslands. Mowing helps counteract vegetation homogenization from land abandonment, but its positive effect diminishes at higher cover of expansive species.

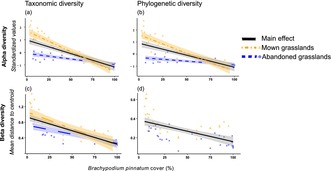

## 1 Introduction

Globally, the escalating human pressure on natural ecosystems is resulting in a profound and accelerated alteration in biodiversity (Díaz et al. 2019; Jandt et al. 2022; Bonari et al. 2025). Understanding mechanisms that drive plant community variation has become crucial for accurately predicting vegetation response to anthropogenic disturbances (Prach and Walker 2011; Staude et al. 2023). Disturbances can significantly shift the dynamics of plant communities by changing the intensity of interspecific competition (Lepš 2014; Fraser et al. 2015; Backhaus et al. 2021). Classical ecology theory posits that plant communities under low disturbance regimes tend to show lower taxonomic diversity, dominated by the presence of few strong competitive species (Grime 2006). On the contrary, increasing disturbance regimes reduce the abundance of these strong competitive species, lowering the intensity of interspecific competition and thus enhancing overall plant diversity (Mayfield and Levine 2010; de Bello et al. 2013; Tardella et al. 2020). While the relationship between disturbances, strong competitive species, and taxonomic diversity is well documented, it fails to provide a broader precise and predictable picture. This is because taxonomic diversity assumes all species are equivalent, overlooking variations in their ecological niches. To address this limitation, phylogenetic diversity offers a valuable approach, as it quantify the amount of niche overlap based on the evolutionary relationships among species. The underlying assumption of phylogenetic diversity is that closely related species are more likely to have higher niche overlap than distantly related ones (i.e., similar multidimensional niche; Anacker and Strauss 2016). In general, the pattern of an undisturbed community dominated by few strong competitors results from the drivers of the ‘weaker‐competitive exclusion’ (Chesson 2000). This process states that only species having higher competitive ability, similar to strong competitors can coexist locally. On the contrary, the increase of disturbance regimes can prevent the establishment of strong competitors, making available previously filled ecological niches for phylogenetically distant species (Letcher et al. 2012). However, disturbance can also act as a filter by selecting only species adapted to the specific nature of the disturbance regime (Zhang et al. 2014). Numerous studies on these topics have compared how disturbance regimes influenced plant communities (e.g., Bonanomi et al. 2006; Catorci, Ottaviani, and Cesaretti 2011; Tardella et al. 2020; Mugnai et al. 2022), leaving largely unexplored the modulating effect of disturbance regimes on the ability of strong competitive species to shape community composition. Understanding the potential role of anthropogenic disturbance in altering the biotic filtering mechanism exerted by strong competitors not only provides theoretical information on assembly rules but can also be translated into management practices aimed at conserving or restoring habitats.

Plant diversity is a multidimensional concept that entails not only different facets (e.g., taxonomic and phylogenetic diversity) but also different spatial components (i.e., alpha or within‐community and beta or between‐communities) (de Bello et al. 2010). Partitioning the diversity into its dimensions may reveal the scale and the extent to which mechanisms of community assembly influence the plant communities. For example, competitive interactions can filter species within the plant communities (alpha diversity), being stronger at this scale (de Bello et al. 2013; Jucker et al. 2013). Also, competitive interaction can influence the degree of similarity between plant communities (beta diversity), since strong competitor species can favour the coexistence of a specific set of species according to their ecological niche. Therefore, accounting for the effects of interspecific competition and disturbance conditions on different spatial components of diversity is essential for unravelling the complexities of community mechanisms.

Semi‐natural grasslands provide an ideal setting for investigating the impacts of disturbance and competition on community assembly due to their rich species diversity at smaller spatial scales (Dengler et al. 2014; Lepš 2014; da Silveira Pontes et al. 2015). Following socio‐economic changes that started after the Second World War, most semi‐natural grasslands across Europe are experiencing the abandonment of traditional land uses, such as mowing. This trend, in turn, is triggering processes that are causing marked variation in plant community (MacDonald et al. 2000; Tälle et al. 2014; Moinardeau et al. 2019; Ferrara et al. 2021; Bonari et al. 2025). In the short term, this variation primarily stems from the proliferation of ‘expansive species’, defined as native species whose local spread and abundance are favoured by recent environmental changes (especially land use) (Axmanová et al. 2024). In the case of semi‐natural grassland, expansive species are, for example, grass species like *Arrhenatherum elatius* , *Brachypodium* spp., *Calamagrostis epigeios, Elymus repens* and *Molinia caerulea* . In semi‐natural grassland, expansive species belong mainly to the same family (Poaceae), sharing similar functional adaptations like tall stature, integrated clonal propagations and high litter deposition, making them strong competitors able to out‐compete many species from local communities with a similar effect of non‐native species (Catorci, Ottaviani, and Cesaretti 2011; Canals et al. 2017; Harásek et al. 2023; Bonari et al. 2025). However, understanding the biotic filters influencing expansive species in abandoned grasslands relies on comparing plant communities under different management conditions, without controlling the potential modulating effect of these different conditions on the relationship between expansive plant species and the plant community. Yet, how variation in management conditions modulates the effect of biotic filters remains largely unknown, mainly because addressing this question requires a complex sampling design in which the expansive species varies across management conditions (e.g., Lepš 2014).

Considering the aforementioned context, this study aims to investigate the effects of management types (i.e., mown and abandoned grasslands), expansive species and their interactions, on alpha and beta taxonomical and phylogenetic plant diversity in semi‐natural grasslands. Overall, assessments of competition largely rely on experimental manipulations involving a limited number of species under controlled environmental conditions, where species biomass or cover represent indicators of competitive performance (Lepš 2014; Conti et al. 2018; Carmona et al. 2019). However, whether the patterns and processes derived from such studies can be extended to the natural communities is largely challenging (La Bella et al. 2024; Hiddink and Davies 2024). Investigating competition in the natural conditions is not straightforward, and causation cannot be strictly claimed due to the co‐occurrence of multiple interacting factors (Conti and Díaz 2013). Nevertheless, using the cover of expansive species as a proxy for their competitive ability, along with diversity indices as indicators of competitive exclusion outcomes, remains the most feasible and informative approach for assessing competition in natural communities (Jucker et al. 2013; Klanderud et al. 2015; Loiola et al. 2018). Specifically, our questions are (i) Does mowing increase alpha and beta taxonomic and phylogenetic diversity compared to abandoned grasslands? (ii) Does an increase in expansive species' cover reduce the alpha and beta taxonomic and phylogenetic diversity of plant communities? (iii) Is the effect of expansive species' cover on plant communities modulated by different management types (i.e., mown and abandoned grasslands)?

## 2 Materials and Methods

### 2.1 Study Area

The study area lies within the central Apennines (Italy), situated in the northern region of the Sibillini Mountains National Park at around 1380 m a.s.l. (43°01′16″ N, 13°13′36″ E) (Appendix S1, Figure S1 in Data S1). The geological composition comprises limestone bedrock, resulting in acid/sub‐acid soil (pH 5.0–6.0) with a total depth ranging from 40 to 50 cm. The prevailing climate is classified as sub‐Mediterranean, characterised by a mean annual temperature that stands at 7.3°C with a four‐month period below 0°C and a likelihood of significant frosts from October through the end of March. Annual rainfall measures around 1400 mm, with an average of 240 mm during the summer, leading to a brief period of water scarcity (Catorci, Ottaviani, and Cesaretti 2011; Rivas‐Martínez et al. 2011).

The landscape of the study area is primarily composed of grasslands with a patchy distribution of forests and shrublands. For centuries, these semi‐natural grasslands underwent traditional pastoral management practices, involving mowing at the end of June, followed by grazing by sheep and cattle. However, in the last decades, grazing and mowing activities have ceased in some portions of these grasslands, while in other sections, grazing stopped but annual mowing has continued. These varying management practices have led to the development of distinct grassland communities, where abandoned grasslands are dominated by the expansive species belonging to the *Brachypodium* genus (Catorci, Ottaviani, and Cesaretti 2011). From a phytosociological point of view, mown grasslands belong to the *Filipendulo vulgaris–Trifolietum montani* association, while abandoned grassland belongs to the *Nardo strictae–Brachypodietum genuensis* association, with a prevalence of *Brachypodium genuense* (DC.) Roem. et Schult (Catorci et al. 2007, 2008) (hereafter *Brachypodium pinnatum* (L.) P.Beauv).

### 2.2 Vegetation Sampling and Phylogeny

We retrieved vegetation data of mown grassland from a previous study in the same study area (Ferrara and Bricca 2023). These data, collected in the growing season of 2020, consist of 31 plots (0.5 × 0.5 m) placed randomly in homogeneous environmental conditions (similar landform, elevation, slope, and north‐facing aspect) but with varying degrees of *Brachypodium pinnatum* cover in regularly mown grassland once a year. During the growing season of 2023, 30 new plots were placed in the nearby abandoned grassland area using the same protocol. In essence, plots (i) shared the same size as those placed in mown grassland (0.5 × 0.5 m), (ii) were placed in similar macro‐ and meso‐environmental conditions (Table 1), (iii) had different degrees of expansive species cover. Species cover was visually estimated by the same observers (quantified in the percentage of the plot's surface) to ensure comparable measurements with Ferrara and Bricca (2023).

**TABLE 1:** Mean, standard deviation (SD) and range of environmental variables for mown and abandoned grassland. Variation of the environmental variables between mown and abandoned grasslands has been tested with an unpaired *t*‐test (all showing significant changes; *p* < 0.01). Before to run the test, aspect was previously converted to a linear scale from 0 (north) to 180 (south) according to Warren (2008).

Environmental variables	Mown grasslands		Abandoned grasslands	
	Mean (SD)	Range	Mean (SD)	Range
Altitude (m.a.s.l.)	1534 (20)	1504–1589	1465 (32)	1418–1517
Aspect (degrees)	333 (21)	290–360	310 (10)	290–329
Slope (°)	5 (2)	2–9	10 (2)	7–15
Litter (%)	9.7 (4.3)	4–20	92 (13)	60–100

The complete species list was used to generate a phylogenetic tree with the most inclusive and updated phylogeny for vascular plants (Smith and Brown 2018). We adopted ‘Scenario 3’ since it is the most conservative approach, avoiding random solutions by adding genera or species as basal polytomies within families or genera (Jin and Qian 2019). All species were resolved, and no polytomies were found. Before building the phylogenetic tree (Appendix S2, Figure S2 in Data S1), species nomenclature has been standardised using the WorldFlora R package (Kindt 2020) that uses the World Flora Online as a backbone (https://www.worldfloraonline.org/). Species nomenclature followed World Flora Online (except in cases where authorship was associated with the taxa). The phylogenetic tree was created with the *V.PhyloMaker* function in the V.PhyloMaker R package (Jin and Qian 2019).

### 2.3 Taxonomic and Phylogenetic Diversity Partitioning

To calculate the alpha and beta taxonomic and phylogenetic diversity, we used Rao's quadratic entropy as it provides a robust framework to compare different facets and spatial components of plant communities (de Bello et al. 2010). The alpha diversity was calculated as follows: $Q=\sum_{i,j}^{s}d_{\mathrm{ij}}p_{i}p_{j}$, where *S* is the number of species, *d*_*ij*_ is the dissimilarity between the *i*‐th and *j*‐th species, *p*_*i*_ and *p*_*j*_ are the proportions of the *i*‐th or *j*‐th species. For alpha taxonomic diversity, *d*_*ij*_ = 1 for every *i* ≠ *j*, otherwise *d*_*ij*_ = 0 for every *i* = *j*. In this case, alpha taxonomic diversity corresponds to the Simpson diversity index: $D=\sum_{i=1}^{s}p_{i}^{2}$. For alpha phylogenetic diversity, *d*_*ij*_ expresses the pairwise distance between the pair of tips of the phylogenetic tree, calculated using its branch length (i.e., phylogenetic cophenetic distance matrix) (Sneath and Sokal 1973). Since the phylogenetic distance matrix potentially has no upper limits, its values were scaled to the range 0–1 to have a comparable measure with taxonomic diversity (de Bello et al. 2010).

Beta diversity was calculated using additive partitioning (i.e., *γ* = mean *α* + *β*) for each couple of plots, resulting in a plot‐pairwise matrix. In this case, gamma represents the total diversity of each couple of plots calculated with the above equation. However, since alpha and beta components are not independent, and beta diversity approaches zero as alpha diversity becomes larger, we applied the correction proposed by Jost (2007), which involves the calculation of 1/(1−*α*) and 1/(1−*γ*) for taxonomic and phylogenetic diversity to avoid biased beta diversity values (details in de Bello et al. 2010).

We calculated alpha and beta taxonomic and phylogenetic using the *RaoRel* function in the cati package (Taudière and Violle 2015). To calculate cophenetic distance, we used the *cophenetic* function in the picante package (Kembel et al. 2010).

### 2.4 Statistical Analysis

Preliminarily, we identified particular species as indicators of management types, performing an indicator species analysis (ISA) using permutation tests with 999 runs (Dufrêne and Legendre 1997). From significant species, we excluded all those with relative abundance lower than 0.6 or with relative occurrence 0.25 (de Cáceres et al. 2012; Bricca, Tardella, Ferrara, Xinfang, et al. 2021). This filtering also acted as a sensitivity analysis, minimising the potential influence of rare or temporally variable species and thereby strengthening the robustness of comparisons among management types. ISA was run with the *multipatt* function in the indicspecies package (de Cáceres and Legendre 2009).

The effect of expansive species on alpha taxonomic and phylogenetic diversity was analysed by running regression models. *B. pinnatum* cover was used as the main predictor variable, together with the management types (categorical variable with two levels: mown and abandoned). More importantly, we included the interaction of *B. pinnatum* cover with management types. Normal distribution of residuals, homogeneity of residuals' variances, and independence of the residuals were graphically evaluated (Zuur et al. 2010). Regarding beta diversity, we first ran multivariate homogeneity of group dispersions using the plot‐pairwise matrix of beta taxonomic and phylogenetic diversity (Anderson et al. 2006). This procedure is largely used to quantify the average distance (i.e., dispersion) of each plot from the centroid for each group (mown vs. abandoned grasslands) in multivariate space (Carmona et al. 2012; Janeček et al. 2013; Bricca, Tardella, Ferrara, Xinfang, et al. 2021; Bricca, Tardella, Ferrara, Panichella, and Catorci 2021). Plots with higher distance values (far away from the centroid) mean higher beta diversity compared to plots with lower distance values (close to the centroid) (Figure S3, Appendix S3 in Data S1). Then we used these distance values (as an indicator of beta taxonomic and phylogenetic diversity) as a response variable in similar models to alpha diversity, where expansive species, management types, and their interaction were predictors. As for alpha diversity, model assumptions were graphically evaluated. Multivariate homogeneity of groups dispersion and linear model were run with the *betadisper* function in the vegan package (Oksanen et al. 2024) and the *lm* function in the stat package.

Finally, we evaluated the relative contribution of expansive species and management types for alpha and beta taxonomical and phylogenetic diversity by applying a hierarchical partition using the *hie.part* function in the hie.part package (Nally and Walsh 2004).

## 3 Results

In mown grasslands, a total of 60 species belonging to 25 families were found, whereas in abandoned grasslands, we documented 50 species belonging to 21 families (Appendix S4, Table S1 in Data S1). The number of indicator species increased from abandoned grasslands (*N* = 9) to mown grasslands (*N* = 12). The indicator species for mown grasslands belonged mostly to Lamiaceae and Fabaceae families, while indicator species in abandoned grasslands belonged to Poaceae and Campanulaceae families (Table 2).

**TABLE 2:** Indicator species of abandoned and mown grasslands, resulting from indicator species analysis performed with 999 permutations on the species composition matrix.

	Species	Family	IndVal
Abandoned grasslands	*Agrostis capillaris*	Poaceae	0.658**
	*Campanula glomerata*	Campanulaceae	0.814**
	*Campanula micrantha*	Campanulaceae	0.493*
	*Cruciata glabra*	Rubiaceae	0.577**
	*Avenella flexuosa*	Poaceae	0.548**
	*Dianthus monspessulanus*	Caryophyllaceae	0.775**
	*Leucanthemum adustum*	Asteraceae	0.548**
	*Luzula campestris*	Juncaceae	0.707**
	*Trifolium alpestre*	Fabaceae	0.758**
Mown grasslands	*Betonica officinalis*	Lamiaceae	0.596**
	*Carex macrolepis*	Cyperaceae	0.783**
	*Festuca circummediterranea*	Poaceae	0.672**
	*Knautia calycina*	Caprifoliaceae	0.757**
	*Luzula multiflora*	Juncaceae	0.596**
	*Potentilla rigoana*	Rosaceae	0.783**
	*Rhinanthus wettsteinii*	Orobanchaceae	0.539**
	*Sedum annuum*	Crassulaceae	0.590**
	*Thesium linophyllum*	Santalaceae	0.565**
	*Thymus longicaulis*	Lamiaceae	0.635*
	*Trifolium montanum*	Fabaceae	0.823**
	*Trifolium pratense*	Fabaceae	0.578*

*Note:* Observed indicator values (IndVal) for each significant species are reported. *p < 0.05; **p < 0.01; ***p < 0.001.

**FIGURE 1:**
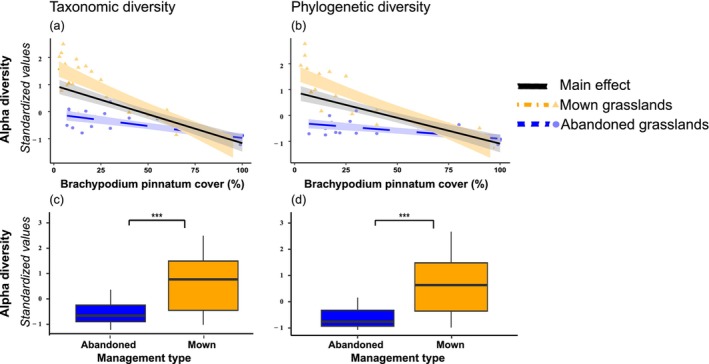
Relationship between expansive species cover (measured as % of ground cover), management type (mown and abandoned) and their interaction, on alpha (a–c) taxonomic and (b–d) phylogenetic diversity. Alpha diversity values have been standardised to zero mean and unit variance to facilitate graphical comparison. Orange triangles and dotdash lines represent mown grassland, blue circles and dotted lines represent abandoned grassland, while continuous black line represents the main effect. Shaded ribbons represents 95% of the confidence interval.

**FIGURE 2:**
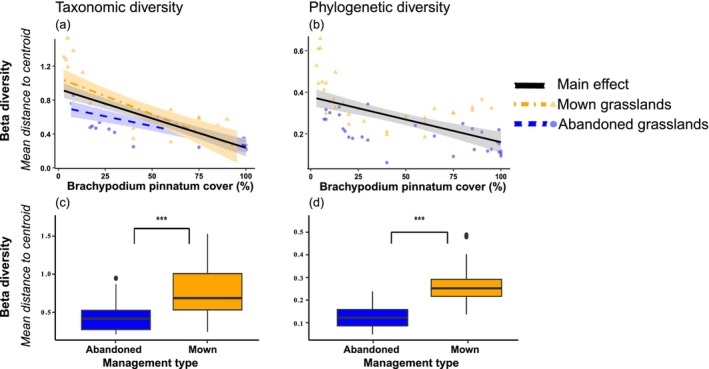
Relationship between expansive species cover (measured as % of ground cover), management type (mown and abandoned) and their interaction, on beta (a–c) taxonomic and (b–d) phylogenetic diversity. Beta diversity values have been standardised to zero mean and unit variance to facilitate graphical comparison. Orange triangles and dotdash lines represent mown grassland, blue circles and dotted lines represent abandoned grassland, while continuous black line reprsents the main effect. Shaded ribbons represents 95% of the confidence interval.

Regarding the analyses on actual values of the diversity indices, overall, our model explained a very high variance (adjusted *R* ^2^), ranging from 56% to 84% (Table 3). For alpha diversity, we found a significant decrease in alpha taxonomical and phylogenetic diversity with the increase of *B. pinnatum* (Figure 1a,b). Similarly, both indices showed a significant effect of management types, with higher values in mown compared to abandoned grassland (Figure 1c,d). Further, we found that alpha taxonomic and phylogenetic diversity was negatively affected by the interaction between *B. pinnatum* cover and management types (Figure 1a,b). Regarding beta diversities, we found a less consistent response for both taxonomic and phylogenetic diversity. Specifically, we found that taxonomic and phylogenetic diversity was significantly affected by *B. pinnatum* cover(Figure 2a,b). Similarly, we found a significant interaction between *B. pinnatum* and management for taxonomic diversity. However, management types alone significantly influenced both diversity components similarly. In essence, mown grassland hosted higher beta taxonomic and phylogenetic diversity (Figure 2c,d).

**TABLE 3:** Detailed results of the linear model for the alpha and beta taxonomic (TD) and phylogenetic (PD) diversity.

Indices	Facet	Brachypodium cover	Management type	Interaction	Adjusted *R* ^2^	AIC
Alpha	TD	−0.02***	3.82***	−0.04***	84***	161
	PD	−0.01**	1.73***	−0.02***	84***	80
Beta	TD	−0.01***	0.34***	−0.01**	67***	−26
	PD	−0.01*	0.15***	−0.01^n.s^.	56***	−148

*Note:* Coefficients of the predictors and model estimates (*R* ^2^ in percentage, and AIC) are reported. Significance of the coefficients and the model is reported as: N.s. *p* > 0.05 (not significant); *p < 0.05; **p < 0.01; ***p < 0.001.

Finally, regarding the hierarchical partitioning analysis, we detected a higher contribution of expansive species compared to management types for all diversity indices, except for beta‐phylogenetic diversity (Table 4).

**TABLE 4:** Absolute (and relative) contribution in percentage (Adjusted *R* ^2^) of *Brachypodium pinnatum* and management conditions for alpha and beta taxonomic and phylogenetic diversity. Independent and joined relative contribution are reported.

Diversity	Facet	*Brachypodium pinnatum*	Management conditions	Joined
Alpha	Taxonomic	48 (67)	24 (33)	10
	Phylogenetic	38 (56)	29 (44)	10
Beta	Taxonomic	51 (79)	14 (21)	8
	Phylogenetic	19 (32)	39 (67)	8

## 4 Discussion

Over recent decades, substantial progress has been made in understanding how environmental conditions shape plant communities (Mayfield and Levine 2010; de Bello et al. 2013). However, the interplay between land management, competitive ability of expansive species, and their combined effects on plant community structure remains insufficiently understood. In this study, we used semi‐natural grasslands as a model system to investigate how expansive species and management regimes (mowing vs. abandonment) affect multiple dimensions of plant diversity, including alpha and beta taxonomic and phylogenetic diversity. Further, we tested if management types modulated the effects of expansive species in influencing the plant community. Our models demonstrated that the effects of management and expansive species varied across spatial components of diversity. Mowing and increasing expansive species cover exerted contrasting effects: the former promoted alpha diversity, while the latter led to its decline. Whereas beta taxonomic diversity was influenced by the types of management and expansive species, beta‐phylogenetic diversity was affected only by management types. Importantly, our findings provide the first empirical evidence that biotic filtering exerted by expansive species can be modulated by management practices.

### 4.1 Management Types

Active management weakens the intensity of interspecific competition, particularly in semi‐natural grasslands where mowing is more detrimental for expansive species because a larger proportion of their phytomass is removed (Klimešová et al. 2011). By decreasing the dominance of expansive species that often monopolise resources in abandoned grassland, mowing allows weaker competitive species to fill previously unavailable ecological niches, enhancing taxonomic diversity (Tälle et al. 2014; Lepš 2014; Bonari et al. 2017; Tardella et al. 2020; Bricca, Tardella, Ferrara, Panichella, and Catorci 2021). Also, our results align with evidence that mowing may counterbalance the homogenisation of taxonomic diversity due to land abandonment even at a larger scale (e.g., Moinardeau et al. 2019; Bricca, Tardella, Ferrara, Xinfang, et al. 2021).

However, as previous research in semi‐natural grasslands has primarily focused on taxonomic responses to mowing, the effects of mowing on phylogenetic dimension were mainly overlooked. By showing that mowing supports the coexistence of distantly related lineages, our results hint that management practices can influence phylogenetic diversity. Mowing contributes to conserving evolutionary potential and functional trait diversity, which are critical for maintaining ecosystem resilience and multifunctionality (Bricca, Tardella, Ferrara, Panichella, and Catorci 2021; Mugnai et al. 2022). This pattern might be attributed to several mechanisms. First, the disturbance caused by mowing prevents successional dynamics that would otherwise favour expansive species and other species similar to expansive ones that belong to fewer, closely related clades (e.g., Poaceae; Lazzaro et al. 2020). Therefore, mowing, by reducing the dominance of expansive species, allows species from a broader phylogenetic lineage to coexist (Zobel 1992). Second, mowing can also act as a selective force promoting the recruitment from seed banks or nearby areas of species with distinct evolutionary histories into the community (Kalamees and Zobel 2002). Thus, mowing might act as an environmental filter that promotes the establishment of species adapted to the specific conditions. As such, in mown grasslands, we found lower cover of the Poaceae family and higher contributions from species belonging to the families of Fabaceae (i.e., *Trifolium* spp.), Caprifoliaceae (i.e., *Knautia calicyna*) and Lamiaceae (i.e., *Thymus longicaulis*). Species found belonging to these families have short size and/or acquisitive strategies, all adaptations to mowing (Bricca et al. 2019). Also, mowing favours greater phylogenetic diversity between plant communities than abandonment, with important implications for ecosystem properties. Greater phylogenetic diversity has been linked to enhanced ecosystem functions, as it often reflects deeper resource‐use complementarity (Flynn et al. 2011; Cadotte et al. 2012). Moreover, the higher phylogenetic diversity may enhance insurance effects (Díaz and Cabido 2001), since species ecologically distinct might better support mid‐ to long‐term potential to cope with environmental changes. In fact, the greater the variation of ecological niches, the lower the number of species required to buffer an ecosystem (Loreau 2000). Thus, our findings suggest that mowing not only influences community composition but may also contribute to the mid/long‐term maintenance of ecosystem properties by preserving phylogenetic breadth. This highlights the potential value of mowing as a biodiversity management tool that supports ecosystem complexity.

### 4.2 Interspecific Competition

We found a similar effect of expansive species in reducing both alpha taxonomic and phylogenetic diversity. In semi‐natural grasslands, such results are expected; numerous studies have documented how the spread of expansive species reduces the diversity of co‐occurring species (Bonanomi et al. 2006; Catorci, Ottaviani, and Cesaretti 2011; Jucker et al. 2013; Mugnai et al. 2022). For example, the development of perennial, compact tussock forms creates a dense matrix that excludes annuals or non‐clonal species, leaving only a few individuals to persist in the gaps between tussocks (Lepš and Buriánek 1990). These effects are further reinforced by high tiller density, frequent branching, and extensive above‐ and belowground clonal growth (Bricca et al. 2020). Moreover, the dominance of tall expansive species alters light availability at ground level, driving the competitive exclusion of shorter species (Catorci, Cesaretti, et al. 2011; Bricca, Tardella, Ferrara, Panichella, and Catorci 2021). Expansive species also tend to produce large amounts of dense litter that accumulate on the soil surface, reducing light penetration and the spatial heterogeneity of microsites, thereby limiting the establishment and growth of seedlings of other species (Janeček and Lepš 2005; Lepš 2014). Overall, these patterns also fit within general ecological theory predicting the homogenisation of local plant communities and the dominance of lineages closely related to strong competitors (Grime 2006; Jucker et al. 2013; Mugnai et al. 2022).

### 4.3 Modulating Effect of Management on Interspecific Competition

Interestingly, the relative intensity of the biotic filtering processes was different across management types, being more marked in mown grasslands compared to abandoned ones. Such a pattern highlights how management types may modulate the role of expansive species in shaping plant communities. Probably, such a modulating effect derives from a distinct spatial scale at which management and competitive interaction took place. For example, management types may represent a primary filter operating on grassland communities, influencing the availability of species. After defining the set of species potentially available (which is higher in mown grasslands), the presence of a large number of weaker competitive species in mown grassland may foster a deeper competitive exclusion effect from expansive species. In contrast, the presence of a lower set of available species and the higher presence of tussock species similar to expansive ones in abandoned grassland may slow down the effect of biotic filtering. We suggest that the effects of weaker competitive exclusion could be less intense when expansive species interact with species belonging to the same family (i.e., Poaceae) because of lower disparity in competitive ability, compared with interactions involving species from different families. While the idea that competition and management types interact together in affecting plant communities is not novel, the modulating effect of management types on competitive interaction on natural communities has been largely untested (Brewer 2011; Erktan et al. 2018). Here, we found that both can jointly contribute to shaping plant communities since the biotic filtering effects exerted by expansive species on taxonomic and phylogenetic diversity occurred both in mown and abandoned grassland communities at different intensities (Erktan et al. 2018; Ferrara and Bricca 2023).

Although we did not directly quantify the effect of competition, our approach is the most suitable considering that we tested ecological hypotheses in natural ecosystems (Jucker et al. 2013; Loiola et al. 2018), where confounding factors may be relevant. For example, the choice of small plot size (0.5 × 0.5 m) represents an appropriate dimension to reveal the role of competitive interaction and minimise abiotic variation within the plot (de Bello et al. 2013). Furthermore, we are aware that the vegetation data on mown and abandoned grasslands came from a three‐year difference. However, the floristic composition we found in mown and abandoned grasslands mirrored that detected more than a decade earlier in the same location for both grasslands (Catorci, Ottaviani, and Cesaretti 2011; Catorci, Cesaretti, et al. 2011). Such floristic consistency, due to the lack of land use change over time, suggests that retrieving plot data for mown grasslands three years before abandoned grasslands does not dramatically alter our main findings.

## 5 Conclusion

This study highlights the influence of management types (i.e., mown and abandoned grasslands), expansive species cover, and their interaction in shaping the taxonomic and phylogenetic diversity of semi‐natural grasslands. By integrating both the alpha and beta components of these diversity facets, we offer a more comprehensive understanding of how these ecological drivers affect community assembly processes in natural ecosystems. Our findings underscore the critical role of mowing as a management practice that in semi‐natural grasslands ensures the persistence of richer plant communities, also in terms of evolutionary heritage, thereby promoting ecosystem multifunctionality. In contrast, an increase in expansive species cover reduces plant diversity, likely due to a weaker competitive exclusion process. However, management types can modulate the effects of biotic filtering, which tends to appear more pronounced under conditions of higher species richness, such as in mown areas.

## Author Contributions

**Alessandro Bricca:** conceptualization (lead), data curation (lead), formal analysis (lead), methodology (lead), writing – original draft (lead). **Giacomo Cangelmi:** writing – review and editing (equal). **Arianna Ferrara:** conceptualization (equal), methodology (equal), validation (equal), writing – review and editing (equal).

## Conflicts of Interest

The authors declare no conflicts of interest.

## Supporting information


**Data S1:** ece372773‐sup‐0001‐supinfo.docx.

## Data Availability

Data and R scripts used to run the analysis are stored in Zenodo Repository at the following link: https://doi.org/10.5281/zenodo.17909385 (Bricca et al. 2025).
