# Correction to “Mowing Modulates the Biotic Filter of Expansive Species”

**DOI:** 10.1002/ece3.73050

**Published:** 2026-02-02

Bricca A. Cangelmi G. Ferrara A. 2026 “Mowing Modulates the Biotic Filter of Expansive Species Ecology and Evolution.” 16 no. 1: e72773. https://doi.org/10.1002/ece3.72773.

In the published article, Figure 1 was incorrect because some graphical elements were missing. The corrected figure is shown below.

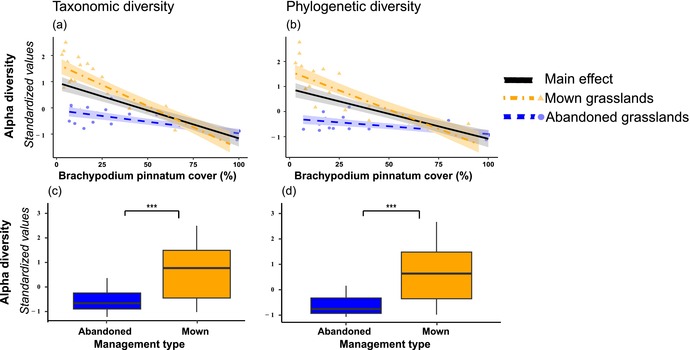

We apologize for this error.

