# Supplementary material for: Mowing Modulates the Biotic Filter of Expansive Species
Source: Ecol Evol. 2026 Jan 5;16(1):e72773. doi: 10.1002/ece3.72773 (PMC12771661; doi:10.1002/ece3.72773)
Supplement: Supplementary file 1 — Data S1: ece372773‐sup‐0001‐supinfo.docx. [file ECE3-16-e72773-s001.docx]

Supplementary materials of “Mowing modulates the biotic filter of expansive species”

**Table of contents**

**Appendix 1** Geographic location of the study area.

**Appendix 2** Phylogenetic tree

**Appendix 3** Plots distribution according to the multivariate homogeneity of group dispersions.

**Appendix 4** Variation of the cover of the families.

**Appendix 1** Geographic location of the study area.


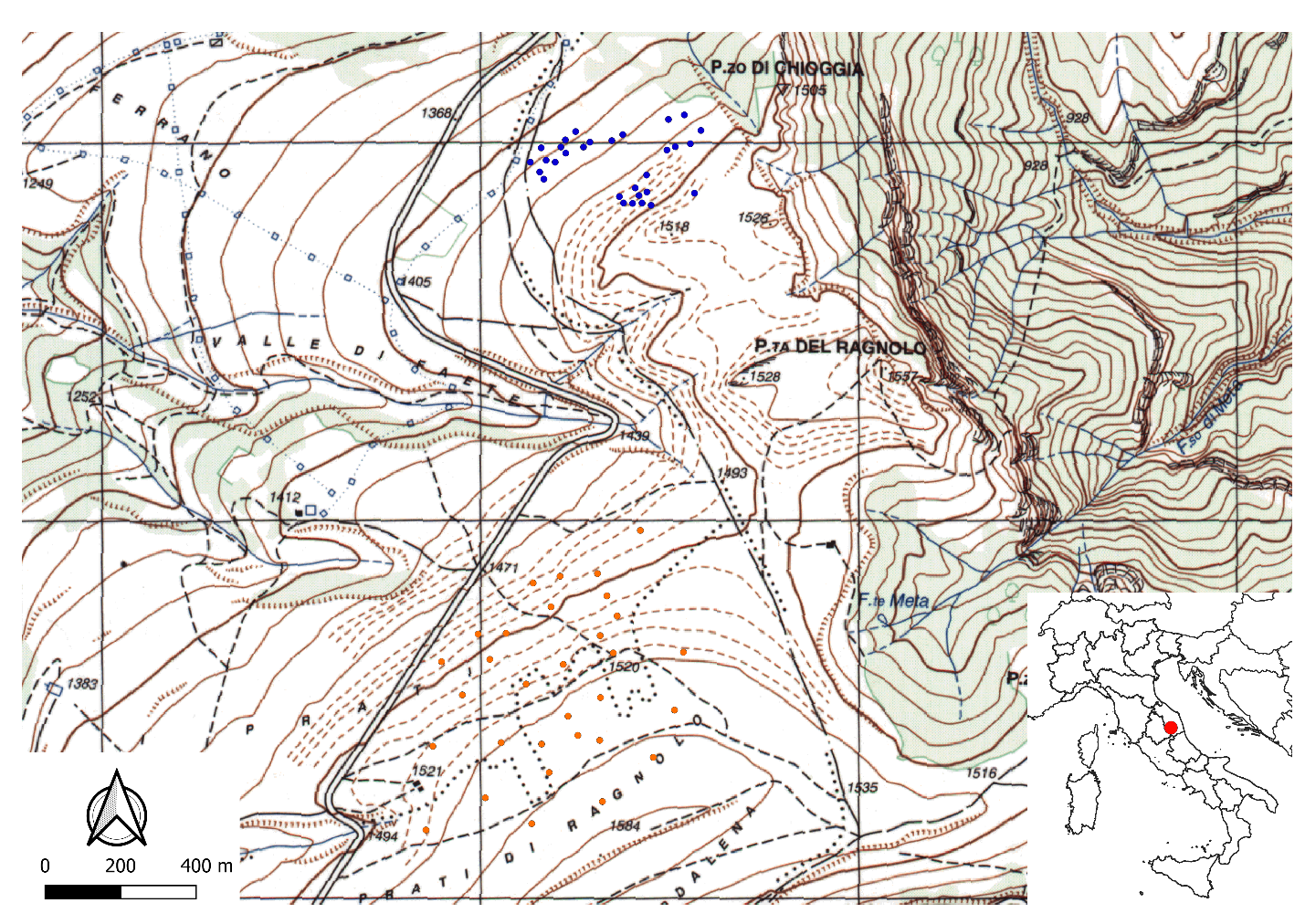


**Figure S1** Locations of the plots in abandoned (in blue) and mowed (in orange) semi-natural grassland under **SPA:** IT5330029 “Dalla Gola del Fiastrone al Monte Vettore” (Central Italy).

**Appendix 2** Phylogenetic tree

**
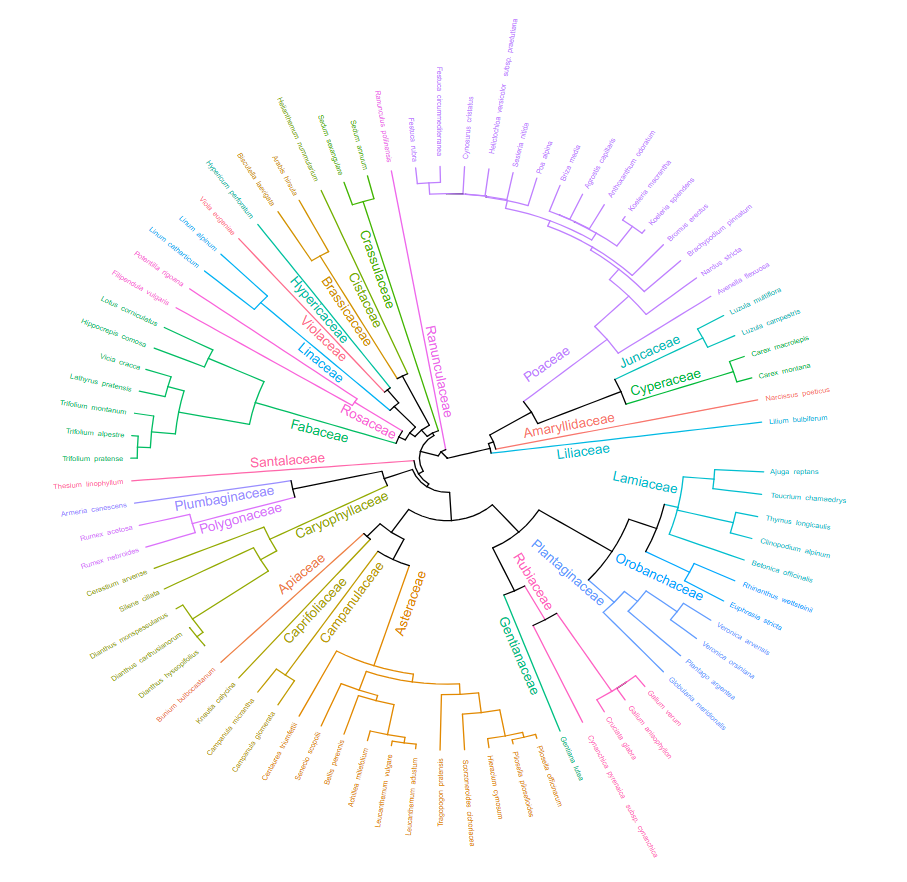
**

**Figure S2** Phylogenetic tree of the recorded species built.

**Appendix 3** Plots distribution according to the multivariate homogeneity of group dispersions.

**
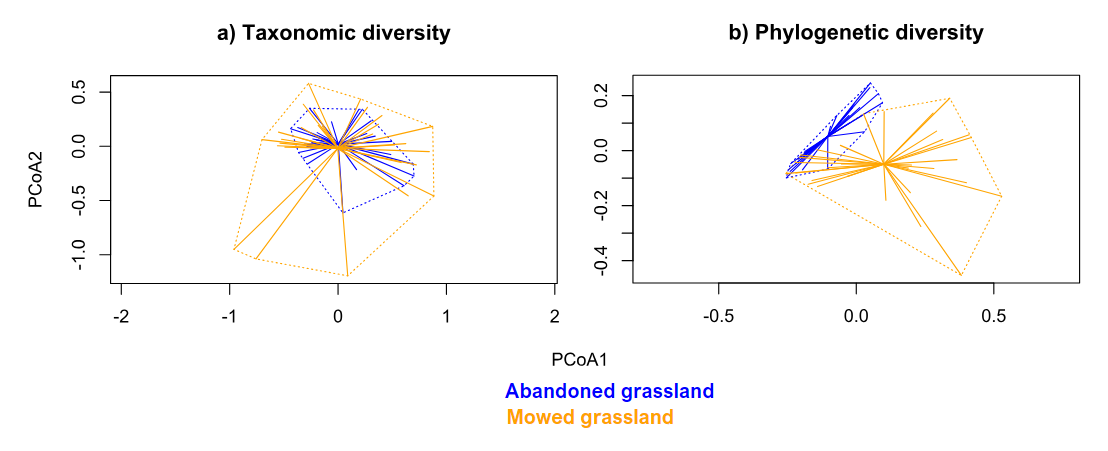
**

**Figure S3** Plots distribution according to the multivariate homogeneity of group dispersions on the plot-pairwise matrix of beta a) taxonomic and b) phylogenetic diversity. The average distance (i.e. dispersion) of each plot from the centroid of each group (mowed vs abandoned grasslands) in multivariate space represents a measure of beta diversity. Plots with high distance value (far away from centroid), means high beta-diversity compared to other plots.

**Appendix 4** Variation of the cover of the families.

**Table S1** Sum of the cover of all the species belonging to the same family across plots for abandoned and mowed grassland.

| **Family** | **Abandoned grassland** | **Mowed grassland** | |
| --- | --- | --- | --- |
| Amaryllidaceae | 0 | 1 |  |
| Apiaceae | 0 | <1 |  |
| Asteraceae | 69 | 100 |  |
| Brassicaceae | 0 | 8 |  |
| Campanulaceae | 12 | 3 |  |
| Caprifoliaceae | 26 | 149 |  |
| Caryophyllaceae | 38 | 22 |  |
| Cistaceae | 10 | 0 |  |
| Crassulaceae | 2 | 10 |  |
| Cyperaceae | 3 | 150 |  |
| Fabaceae | 116 | 324 |  |
| Gentianaceae | 0 | 5 |  |
| Hypericaceae | 2 | 1 |  |
| Juncaceae | 22 | 10 |  |
| Lamiaceae | 3 | 84 |  |
| Liliaceae | 0 | 5 |  |
| Orobanchaceae | <1 | 9 |  |
| Plantaginaceae | 8 | 20 |  |
| Plumbaginaceae | <1 | <1 |  |
| Poaceae | 2714 | 1937 |  |
| Polygonaceae | <1 | 12 |  |
| Ranunculaceae | 2 | 9 |  |
| Rosaceae | 40 | 103 |  |
| Rubiaceae | 17.3 | 24 |  |
| Santalaceae | <1 | 28 |  |
| Violaceae | 2 | 6 |  |
